# The Value of Intravoxel Incoherent Motion Diffusion-Weighted Magnetic Resonance Imaging Combined With Texture Analysis of Evaluating the Extramural Vascular Invasion in Rectal Adenocarcinoma

**DOI:** 10.3389/fonc.2022.813138

**Published:** 2022-03-03

**Authors:** Fei Gao, Bin Shi, Peipei Wang, Chuanbin Wang, Xin Fang, Jiangning Dong, Tingting Lin

**Affiliations:** Department of Radiology, The First Affiliated Hospital of University of Science and Technology of China (USTC), Division of Life Sciences and Medicine, University of Science and Technology of China, Anhui Provincial Cancer Hospital, Hefei, China

**Keywords:** rectal Carcinoma, extramural vascular invasion, intravoxel incoherent motion, diffusion-weighted imaging, magnetic resonance imagine, texture analysis

## Abstract

**Purpose:**

This study aims to evaluate the value of 3.0T MRI Intravoxel Incoherent motion diffusion-weighted magnetic resonance imaging (IVIM-DWI) combined with texture analysis (TA) for evaluating extramural vascular invasion (EMVI) of rectal adenocarcinoma.

**Methods:**

Ninety-six patients with pathologically confirmed rectal adenocarcinoma after surgical resections were collected. Patients were divided into the EMVI positive group (n=39) and the EMVI negative group (n=57). We measured the IVIM-DWI parameters and TA parameters of rectal adenocarcinoma. We compare the differences of the above parameters between the two groups and establish a prediction model through multivariate logistic regression analysis. the ROC curve was performed for parameters with each individual and in combination.

**Results:**

ADC, D, D* value between the two groups were statistically significant (*P*= 0.015,0.031,0). Six groups of texture parameters were statistically significant between the two groups (*P*=0.007,0.037,0.011,0.005,0.007,0.002). Logistic regression prediction model shows that GLCM entropy_ALL DIRECTION_offset7_SD and D* are important independent predictors, and the AUC of the regression prediction model was 0.821, the sensitivity was 92.98%, the specificity was 61.54%, and the Yoden index was 0.5452. The AUC was significantly higher than that of other single parameters.

**Conclusion:**

3.0T MRI IVIM-DWI parameters combined with texture analysis can provide valuable information for EMVI evaluation of rectal adenocarcinoma before the operation.

## Introduction

Colorectal cancer is one of the common gastrointestinal tumors. The extra-mural vascular invasion (EMVI) refers to the outside muscularis propria rectum intravascular tumor cells. Previous studies have confirmed that MRI-detected extramural vascular invasion is an independent prognostic factor for synchronous metastasis in patients with rectal cancer (1). It is one of the important factors of poor prognosis of rectal cancer and is considered to be an important factor that causes local recurrence, lymph node metastasis, and distant metastasis of tumor (2, 3). The identification of EMVI is very important for choosing the best treatment for rectal cancer. Magnetic resonance imaging (MRI) is the standard method for preoperative evaluation of rectal cancer by EMVI, which has high sensitivity and medium specificity, and is based on the size of the tumor and the location of the corresponding blood vessels (4). However, high-resolution magnetic resonance imaging can only provide limited morphological information and can’t represent all the information of tumor heterogeneity. Therefore, we want to explore some more accurate methods to evaluate the role of EMVI in rectal carcinoma. Intravoxel incoherent motion (IVIM) is a method to evaluate diffusion-weighted imaging (DWI) with the use of multiple b values to separate the contribution of perfusion from tissue diffusion (5), which may provide more information for predicting and evaluating EMVI of rectal cancer. Texture analysis (TA) extracts texture features that cannot be recognized by the naked eyes by computer software, and can quantify the gray scale, space and structure of MRI image pixels. The obtained parameters can be used to quantitatively evaluate the heterogeneity of different lesions, assess the lesions microenvironment, and evaluate the curative effect after tumor treatment (6, 7). This study intends to conduct a retrospective analysis of IVIM-DWI parameters and TA characteristics in both negative and positive groups of rectal adenocarcinoma. In addition, the predictive value of IVIM-DWI combined with quantitative parameters of TA parameters based on MRI in preoperative EMVI rectal adenocarcinoma was explored.

## Materials and Methods

### Patients and Clinical Information

This retrospective study was approved by the institutional review committee, and the requirement of informed consent of patients was waived. A total of 96 patients with rectal adenocarcinoma from June 2018 to December 2020 were collected in a retrospective way, including 59 males and 37 females (age 60.1 ± 11.1). The patients received MRI and IVIM-DWI, who underwent radical resection within 1 month after the MRI scan. 39 patients with positive EMVI and 57 patients with negative EMVI confirmed by pathology after surgery. Inclusion criteria: (1) the tumor of the patient was confirmed as rectal adenocarcinoma by surgery and pathology. (2) the pathological report included EMVI. (3) the patient did not receive any non-operative treatment, such as radiotherapy or chemotherapy, before the operation. (4) the patient is scanned on the same MRI platform with a unified imaging protocol. (5) the image quality was adequate for diagnosis and measurement. Exclusion criteria: (1) incomplete pathological result(n=5).(2)previous treatment(radiation or chemotherapy)(n=20). (3) MRI contraindications or MRI quality that cannot meet the diagnostic requirements(n=2). (4)Poor MRI image quality or IVIM-DWI scanning sequence is incomplete(n=2). The patient selection process is shown in **Figure S1**.

### Imaging Protocol

MRI examinations were performed on a 3.0-T system (Signa HDXT, General Electric Healthcare, Waukesha, WI, USA), which was equipped with an eight-channel torso array coil. All patients were scanned by conventional sequence and IVIM-DWI with multiple b values. Patients was placed in the supine position and breathed freely during collection. In the chemical shift selective saturation (CHESS) sequence, the axial IVIM-DWI with FS was obtained by using single-shot echo-planar imaging (EPI) pulse sequence, which has 10 b values (0, 10, 20, 50, 100, 200, 400, 800, 1,200 and 1,500 s/mm^2^), repetition time (TR) = 4000 ms, echo time (TE) =75 ms, FOV 42 cm×42 cm, matrix 96×130, slice thickness/gap: 4 mm/1 mm, number of excitations (NEX) = 6. Liver acquisition with a volume acceleration (LAVA) sequence was used for contrast-enhanced pelvic imaging, and a high-pressure injector (Optistar LE; Mallinckrodt; Cincinnati, Ohio, USA) was used for injection of contrast material (0.1 mmol/kg, Gadodiamide, General Electric Healthcare, USA). The details of the LAVA MR parameters are as follows: TR=4.5 ms, TE =1.3 ms, flip angle =15°, NEX = 1, band width =166.67 kHz. These images were obtained from multiple phases after injection of the contrast agent in the axial and sagittal planes (postcontrast at 20 s, 60 s, and 120 s in axial planes and 150 s in sagittal planes).

### Radiologic Evaluation

Two radiologists with more than 10 years’ experience in MRI diagnosis analyzed and measured the images. They were blind to the pathological results, and discrepancies were resolved by consensus. Using GE ADW 4.6 workstation, Function tool-MADC software was used to measure IVIM-DWI parameters. The largest area of horizontal tumor was selected for measurement. For the region of interest (ROI), the areas with homogeneous signal intensities were selected and round areas (approximately 25-30 mm^2^) were drawn manually on the parametric maps by two radiologists. The ROIs drawing should avoid the areas of susceptibility artifacts, large necrosis and cystic areas and hemorrhage. The same level was measured three times and the mean value was obtained. The parameters measured by IVIM-DWI included the following: apparent diffusion coefficient(ADC), pure diffusion coefficient(D), pseudo diffusion coefficient (D*), and perfusion fraction(f).

**Figure 1:**
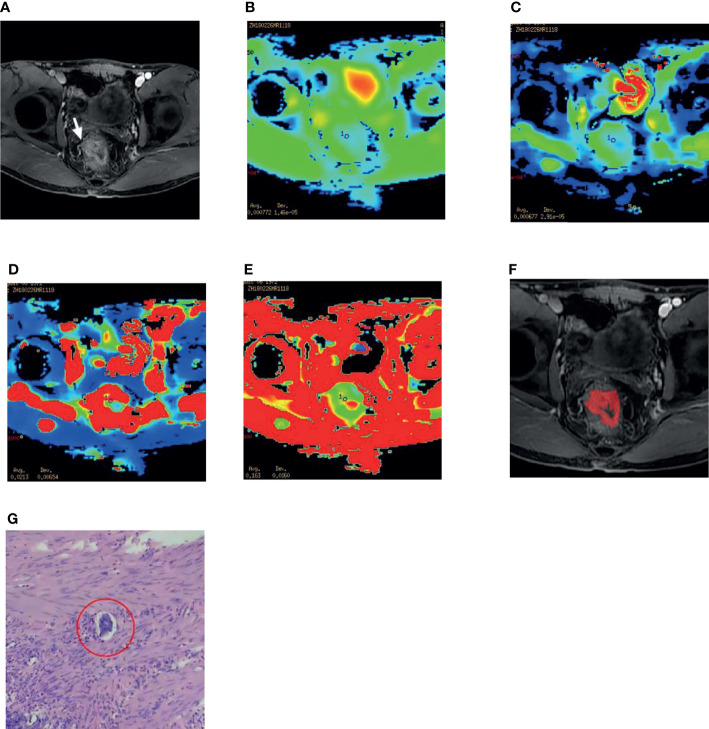
A 45-year-old male patient with rectal adenocarcinoma. **(A)** The right lateral wall of the rectum was invaded, and the origin of the vessels was changed (white arrow). **(B–E)** After selecting the ROI on the DWI image, the IVIM-DWI parameters ADC,D,D* and f value were obtained. **(F)** Texture analysis, The radiologist mapped the ROI on the maximum size of the lesion. **(G)** The postoperative specimen of this patient is confirmed by pathology to be differentiated Adenocarcinoma of Rectum and EMVI positive, ulcerated, and the red circle shows intravascular cancer embolus (HE 400).

All patients’ magnetic resonance images were exported from PACS workstation in DICOM format. The largest area of level tumor of enhanced venous phase (postcontrast at 60 s in axial planes)was selected to measure for Texture analysis, and we used the ITK-SNAP software to sketch the ROI. At the same time, the IVIM-DWI images were referenced. After the discussion of two senior attending radiologists, ROI was determined, and the tumor boundary was drawn manually. The ROI should cover the whole tumor as much as possible on the largest area of the tumor (**Figure 1**). Texture analysis parameters were obtained after dimension reduction and feature screening by the A.K software(Analysis Kit, Kinetics Version 2.1, GE Healthcare) (**Table S1**). 197 texture features were extracted, and the lasso algorithm was used to reduce dimension and select features, and the results were obtained.

### Histopathology Evaluation

All the patients underwent total mesorectal excision, which was confirmed by pathology after operation. According to the definition of EMVI in pathology (8), the positive criteria for EMVI were as follows: tumor cells were observed by HE staining to encase and invade the adipose tissue around the intestinal wall or the vascular wall under the serous membrane, or tumor cells invaded the vascular lumen outside the intestinal wall to form tumor cell emboli.

### Statistical Analysis

We used SPSS statistical analysis (SPSS 22.0, IBM Corporation, Armonk NY, USA) and MedCalc(16.8.4 version) software, and the intraclass correlation coefficients (ICCs) were used to evaluate the consistency between interobserver in IVIM-DWI and TA parameters measurement (ICC >0.75 means almost complete consistency). Then, the normal distribution of IVIM-DWI parameters and TA parameters were tested. The values that conform to the normal distribution are represented by $\bar{x}\pm s$ , and those that do not conform to the normal distribution are represented by median (quartile). According to the characteristics of data distribution, an independent sample T-test or non-parametric test (Mann-Whitney U rank-sum test) was used for comparative analysis, and *P* < 0.05 was statistically significant. Using the Receiver Operating Characteristic (ROC) curve analysis, we choose the maximum Yoden Index as the critical value, and calculate the area under the curve (AUC, 95% ci) as a valuable parameter, sensitivity, specificity, so as to evaluate the diagnostic effect of these parameters on EMVI negative and positive rectal adenocarcinoma.

Finally, statistical significance parameters are included in multiple logistic regression analysis, EMVI differential diagnosis model of rectal adenocarcinoma is established, and ROC curve analysis was used to obtain the diagnosis effect of the predictive model. Z test was carried out for the prediction model and the ROC curve of a single parameter in IVIM-DWI and TA.

## Results

The results showed that the two radiologists have good inter-observer consistency in IVIM-DWI parameters and TA parameters, and the ICCs is between 0.81 and 0.90.

**Figure 2:**
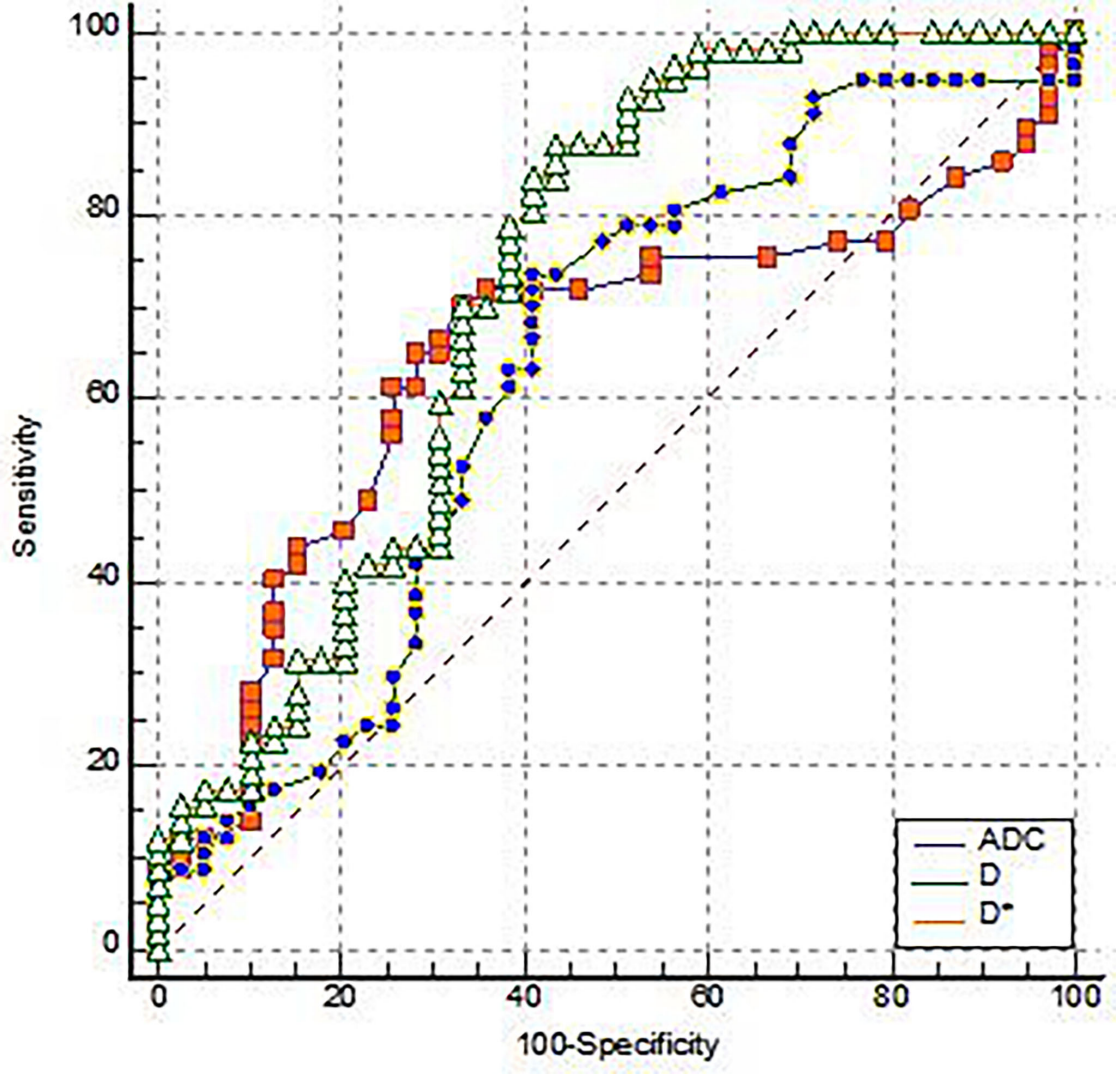
The ROC analysis for the IVIM-DWI parameters of the EMVI positive group and the EMVI negative group.

ADC, D, and D* of the EMVI positive group were 0.91(0.15)×10^-3^ mm^2^/s, 0. 67(0.35)×10^-3^ mm^2^/s and 13.5(27.62)×10^-3^ mm^2^/s, respectively. The negative group parameter was 0.78(0.27)×10^-3^ mm^2^/s, 0.53(0.23)×10^-3^ mm^2^/s, 5.33(4.43)×10^-3^ mm^2^/s, there was statistical significance between the two groups (**Table 1**). Among them, the AUC of D* is the highest (0.731), and its sensitivity and specificity were 87.72% and 56.41%, respectively. The Yoden index was 0.4413 (**Figure 2**).

**Table 1:** Statistical results of IVIM-DWI parameters between the EMVI positive group and the EMVI negative group of rectal adenocarcinoma.

	n	ADC (10^-3^mm^2^/s)	D (10^-3^mm^2^/s)	D* (10^-3^mm^2^/s)	f(%)
positive	39	0.91 (0.15)	0.67 (0.35)	13.5 (27.62)	36.86 ± 16.01
negative	57	0.78 (0.27)	0.53 (0.23)	5.33 (4.43)	37.34 ± 16.07
t(Z)		-2.436	-2.163	-3.831	-0.141
*P*		0.015	0.031	0	0.612

The f values of two groups were normal distribution and analyzed by independent sample t test, P < 0.05 was statistically significant, and t was the non-parametric rank sum-test. The ADC, D and D* values of the two groups did not conform to normal distribution and were analyzed by non-parametric test (Mann-Whitney U rank-sum test). P < 0.05 was statistically significant, and Z was the statistic obtained by non-parametric rank-sum test.

ADC, apparent diffusion coefficient; D, pure diffusion coefficient; D*, pseudodiffusion coefficient; f, perfusion fraction.

The 197 texture features extracted from the image are selected by dimensionality reduction, and six groups of texture parameters are obtained as follows: histogramEnergy, MeanDeviation, uniformity, Correlation_AllDirection_offset4_SD, GLCMEntropy_AllDirection_offset4_SD, GLCMEntropy_AllDirection_offset7_SD. All these six parameters have statistical significance between EMVI positive and negative groups (*P* < 0.05) (**Table 2**).

**Table 2:** Statistical results of TA between the EMVI positive group and the EMVI negative group of rectal adenocarcinoma.

	n	Histogram Energy	Mean Deviation	uniformity	Correlation_AllDirection_offset4_SD	GLCMEntropy_AllDirection_offset4_SD	GLCMEntropy_AllDirection_offset7_SD
positive	39	0.0114193(0.00283)	-0.00000000163(0.000000369)	0.838177(0.0444)	0.0000000136(0.000000022)	0.0066762(0.01291)	0.030205(0.06736)
negative	57	0.0105037(0.00221722)	0.000000094(0.0000004)	0.819747(0.0365765)	0.00000001(0)	0.00427244(0.0072046)	0.0175849(0.0281982)
Z		-2.719	-2.081	-2.533	-2.779	-2.719	-3.174
*P*		0.007	0.037	0.011	0.005	0.007	0.002

All texture parameters in the two groups did not conform to normal distribution, and non-parametric test (Mann-Whitney U rank-sum test) was used for analysis. Median(quartile) was expressed, P <0.05 was statistically significant, and Z was the statistic obtained by non-parametric rank-sum test.

**Figure 3:**
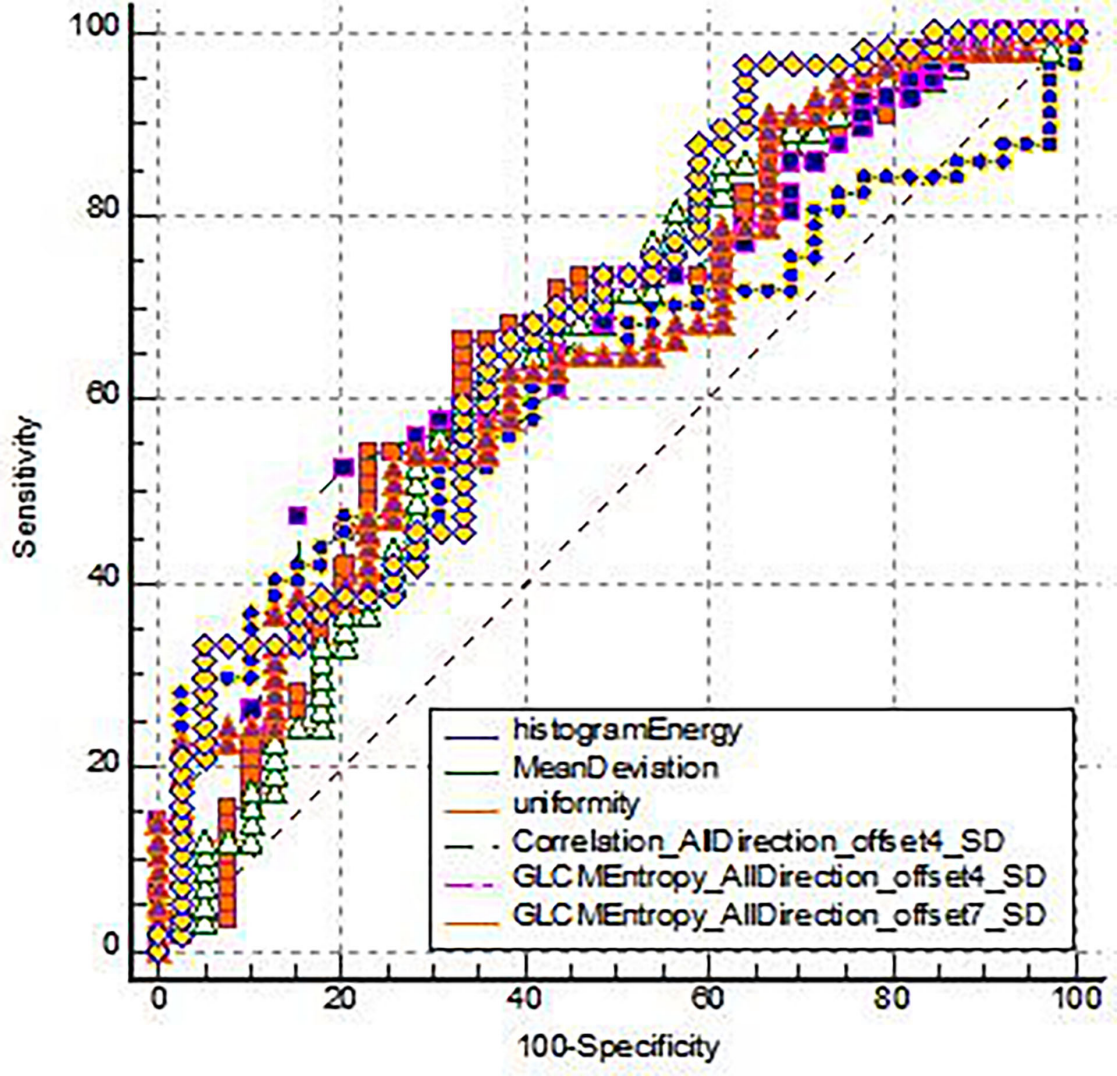
The ROC analysis for the TA parameters of the EMVI positive group and the EMVI negative group.

The diagnostic efficacy of IVIM-DWI and TA parameters was compared (**Table 3**). The maximum AUC of ROC of GLCMEntropy_AllDirection_offset7_SD is 0.691. The sensitivity and specificity of this parameter were 96.49% and 35.90%, respectively. The Yoden index was 0.3239 (**Figure 3**).

**Table 3:** The ROC analysis for each individual feature, and IVIM-DWI combined with TA.

parameters	AUC(95% ci)	Sensitivity(%)	Specificity(%)	Yoden index
ADC(10^-3^mm^2^/s)	0.649(0.545~0.743)	70.18	66.67	0.3684
D(10^-3^mm^2^/s)	0.634(0.529~0.730)	73.68	58.97	0.3266
D*(10^-3^mm^2^/s)	0.731(0.631~0.816)	87.72	56.41	0.4413
histogramEnergy	0.664(0.560~0.757)	66.67	66.67	0.3333
MeanDeviation	0.626(0.521~0.722)	40.35	87.18	0.2753
Uniformity	0.653(0.549~0.747)	63.16	64.10	0.2726
Correlation_AllDirection_offset4_SD	0.674(0.571~0.766)	52.63	79.49	0.3212
GLCMEntropy_AllDirection_offset4_SD	0.664(0.560~0.757)	52.63	79.36	0.2699
GLCMEntropy_AllDirection_offset7_SD	0.691(0.589~0.782)	96.49	35.90	0.3239
D* combined with TA	0.821(0.730~0.892)	92.98	61.54	0.5452

**Figure 4:**
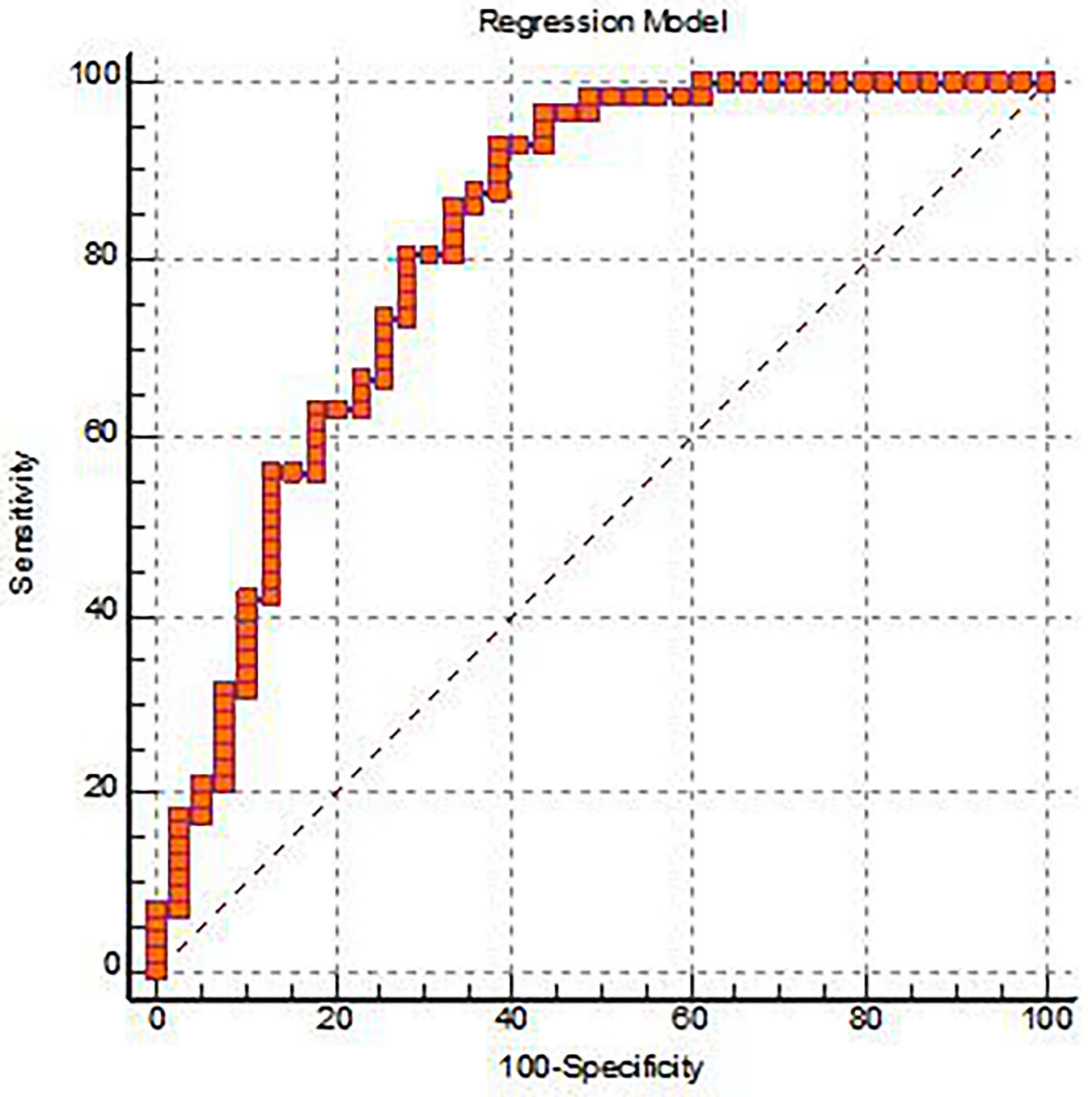
The ROC analysis for the Logistic regression prediction model of the EMVI positive group and the EMVI negative group.

In this study, all independent variables with statistically significant differences between groups were included in logistic regression, and we found that GLCMEntropy_AllDirection_offset7_SD(OR = 0, *P* = 0.003)、D*(OR = 0, *P* = 0)were important independent predictors, and a multivariate logistic regression diagnosis model was established. The AUC, sensitivity, specificity, and Yoden index of the ROC curve were 0.821, 92.98%, 61.54%, and 0.5452, respectively (**Figure 4** and **Table 3**). Delong.test was performed on the ROC curves of the prediction model, IVIM-DWI, and TA, which showed that the AUC of the prediction model was significantly higher than that of other single parameters (*P* < 0.05).

## Discussion

### Application of IVIM-DWI Parameters in EMVI Evaluation of Rectal Adenocarcinoma

The basic principle of IVIM-DWI imaging is that multiple b values are applied to DWI sampling, and the standard ADC, D, D* and f values through double-exponential fitting analysis. Different from the traditional single-index DWI sequence, the IVIM-DWI sequence can show water diffusion and microcirculation perfusion, when b<200 s/mm^2^, It reflects the information of microcirculation perfusion, when b>200 s/mm^2^, it reveals the diffusion movement of pure water molecules. Therefore, IVIM-DWI can quantify the diffusion coefficient and microcirculation perfusion information accurately. More information can be provided for clinical diagnosis (9, 10).

In this study, ADC value and D value show that the diffusion of water molecules in the two groups is obviously limited, which was consistent with the biological signs of malignant adenocarcinoma, such as higher cell density, less free water molecules in the tumor cells, smaller extracellular space and so on. D value excluded the influence of blood perfusion, could more accurately reflect the diffusion ability of simple water molecules in tissue cells, and accurately provide the proliferation information such as nuclear atypia and nuclear-plasma ratio of tumor cell. In this study, ADC values and D values in the positive EMVI group are higher than those in the negative group, with a statistically significant difference between the two groups (*P <*0.05). which is consistent with the Sun et al. study (11). The author analyzed the reasons and found that the positive groups had higher pathological T staging (≥T3), and the tumor tissue would break through the Lamina propria and might invade the mesorectum, Therefore, the tumor cells in the positive group had more growth space than the negative group, and the water molecules in the positive group were less restricted than those in the negative group. In the negative group, the stage of tumor tissue was T2 or T1, and the tumor was confined to the intrinsic muscularis propria but had not yet broken through the intrinsic muscularis, so the growth space of tumor cells was relatively limited. The density of tumor cell was large, the proportion of nuclear plasma was high, and the diffusion of water molecules was obviously limited.

In this study, D* value in the EMVI positive group were higher than those in the negative group, and the difference between the two groups is statistically significant (*P <*0.05). The f value between the two groups was not statistically significant (*P* > 0.05), and there were few research records. Surov et al. (12) reported that D* was positively correlated with the grade of rectal cancer. On the contrary, f value was not correlated with the grade of the tumor, which was coarsely consistent with this study. D* and f values reflect the information of microcirculation perfusion in the selected voxel, in which D* value represents the diffusion effect of microcirculation perfusion in the voxel, and D* value reflects the microcirculation blood perfusion in the capillary network, and D* value is related to the average vessel diameter of the microvessels. F value represents the volume percentage of microcirculation perfusion effect in voxel in the overall diffusion effect, reflecting the blood volume of capillaries, and has a good correlation with the total area and the number of blood vessels. Because of the proliferation of tumor cells and angiogenesis of rectal adenocarcinoma, the new tumor blood vessel grows too fast, showing an irregular shape, increasing average diameter of capillary, resulting in a large number of immature new blood vessels; Therefore, it reflects the ability of tissue perfusion D* value rise with the increase of tumor malignant degree, and tumor malignancy degree will have a certain positive correlation (13). When evaluating the diagnostic efficacy of IVIM-DWI parameters by ROC curve, AUC of D* value was the highest, which fully proved that D* value was significantly different between the negative and positive EMVI groups and could be used as an important index for differential diagnosis. In this study, we found that there was no statistical difference in f value, which reflected the microcirculation blood volume between the two groups. It can be concluded that f value was the volume percentage of the perfusion effect to the total diffusion effect, and f value was influenced by perfusion and diffusion of common tumor tissue, and was related to tumor tissue proliferation, cell density, the pure diffusion of water molecules, the number of new blood vessels, total area of tumor angiogenesis, microcirculation blood flow velocity, etc. The value of f was influenced by many factors, so there was no statistical difference in this group.

### Application of TA in EMVI Evaluation of Rectal Adenocarcinoma

Radiomics aims to translate medical images into quantitative data, defined as biomarkers, which may reveal a deeper level of detail than that, which is accessible to the unaided human eye, so as to quantify tumor phenotypes, which could aid in clinical decision-making (14).Radiomics may provide quantitative and objective support for decisions surrounding cancer detection and treatment (15, 16).TA belongs to radiomics. TA by mathematical methods of quantitative imaging image pixel gray level statistics and spatial distribution and structure information, to extract the texture feature which cannot be identified by the naked eye, revealing the heterogeneity of tumor histologic features and certain genes, and by using the quantitative information of the differential diagnosis of the disease, grading, classification and evaluation of curative effect (7, 17). At present, TA technology has been widely used in the diagnosis, effect evaluation and prognosis prediction of tumors in the brain, lung, breast, liver and pelvic cavity (18–21). It is mainly used in the staging of rectal cancer and the evaluation of neoadjuvant chemotherapy, etc. However, very few studies are documented on EMVI in rectal cancer (22). Texture features extracted by texture analysis include: first-order features, which is also known as histogram analysis. It describes the grayscale distribution of individual pixel values in the ROI, including MeanValue, variance, skewness, kurtosis and entropy. Second-order characteristics can calculate two adjacent pixels and local texture feature, commonly used method including gray level co-occurrence matrix (GLCM) and gray-level run-length matrix (GLRLM), etc. The higher-order feature uses statistical method to analyze the local image information.

In this study, six texture features are finally selected, including HistogramEnergy, MeanDeviation, Uniformity, Correlation_AllDirection_offset4_SD, GLCMEntropy_AllDirection_offset4_SD, and GLCMEntropy_AllDirection_offset7_SD. The histogram energy is the voxel value in the image, and a larger value means a larger sum of the squares of these values. Correlation is one of GLCM features, which indicates the linear correlation between gray value and their respective voxels in GLCM. GLCM entropy refers to the uncertainty or randomness in the image value. A higher entropy value means that the distribution of elements within the image is scattered and disordered, which indicates that the more complex the texture information within the tumor, the higher the heterogeneity of the tumor. In this study, the EMVI positive value of four texture feature parameters (HistogramEnergy, Correlation_AllDirection_offset4_SD, GLCMEntropy_AllDirection_offset7_SD) is higher than that of the EMVI negative group, indicating that tumors in the EMVI positive group have more heterogeneous biological behaviors. Image texture distribution is uneven, and texture information is more complex and invasive, which accords with the biological characteristics of EMVI-positive tumors with higher malignant degree. The MeanDeviation is one of the first-order features, which refers to the average distance between all intensity values and the average of the image array. Uniformity is also a first-order feature, which is a measure of the uniformity of the image array, in which greater uniformity means a smaller range of discrete intensity values. In this study, the MeanDeviation value of EMVI positive deviation is lower than that of EMVI negative group, and the Uniformity is greater than that of EMVI negative groups, which promotes the even distribution of the image pixel in the EMVI positive group. The author analyzed that when TA was measured in venous phase in this case, EMVI positive tumor was larger than EMVI negative tumor, and there were more venous drainage vessels in the phase, and the venous images were uniform, which might be the reason for this result.

The difference of 6 texture parameters between EMVI positive group and EMVI negative group was statistically significant, which indicated that texture analysis can provide quantitative information for the discrimination between negative and positive EMVI. Liu et al. (23) analyzed the correlation between the texture characteristics of ADC map and rectal cancer stages and found that entropy could be used as an independent predictor of outer wall invasion, suggesting that entropy-based on ADC map texture characteristics was also a significant assessment of rectal cancer EMVI, which was consistent with the conclusion of this study. In addition, among the six texture analysis parameters to evaluate the EMVI positive of rectal adenocarcinoma, the area under the ROC curve of GLCMEntropy_AllDirection_offset7_SD was the highest (0.691), and its sensitivity and specificity were 96.49% and 35.90%, respectively, which indicates that the diagnostic efficiency of correlation is high. Therefore, the value of MRI texture analysis in EMVI positive diagnosis of rectal adenocarcinoma is confirmed.

### The Predictive Significance to Combine Values of IVIM-DWI and TA in EMVI of Rectal Adenocarcinoma

The parameters calculated by IVIM-DWI using the double-exponential diffusion attenuation model can reflect the diffusion of water molecules in tissues and capillary microcirculation perfusion (24). Although the organization’s information is reflected from the molecular level, the information provided is still limited. TA can extract a large number of texture features that cannot be recognized by the naked eye to reflect inter-tissue heterogeneity, which has become a hot spot in medical and scientific research. IVIM-DWI and TA have been applied to preoperative assessment of rectal cancer and have shown some predictive value and potential for clinical application. (25–27). However, the diagnostic value of IVIM-DWI combined with TA for EMVI of rectal adenocarcinoma has not been reported. In this study, IVIM-DWI and TA are combined, and these two valuable parameters are brought into the logistic regression analysis, and a multivariate logistic regression analysis diagnosis model was constructed. From this, it was concluded that GLCMEntropy_AllDirection_offset7_SD and D* were two important independent predictors. In this study, GLCMEntropy_AllDirection_offset7_SD can reflect the heterogeneity of tumor and is an important parameter to distinguish negative and positive EMVI, while D* can reflect the microcirculation perfusion of the capillary network, which is of great significance in the differential diagnosis. In addition, this study established the multi-factor logistic regression diagnosis model of AUC is 0.821, the sensitivity of 92.98% and 61.54%, about an index is 0.5452, the AUC value and an index were greater than IVIM-DWI and any other parameters in the TA. This shows that the diagnostic efficiency is higher, and it reflects that the IVIM-DWI and TA are effective combined diagnostic methods for EMVI. The combination of IVIM-DWI and TA can better reflect the pathology and micromorphology of rectal Adenocarcinoma, and further improve the ability of preoperative prediction EMVI of rectal Adenocarcinoma.

### Research Deficiencies and Prospects

There are still many limitations in this research. Firstly, the number of patients enrolled in this study is not large. Therefore, it is necessary to increase the sample size in future research. Secondly, the ROI selection of IVIM only selects the part of the limited diffusion areas that cannot contain all the tumor information at this level. In addition, the ROI selection of TA parameters was selected and measured at the two-dimensional level, which fails to reflect the tumor imaging omics information in the three-dimensional full volume. In future studies, we will continue to increase the sample size and improve the data measurement method, so as to provide more accurate and objective quantitative analysis data for EMVI differentiation of rectal adenocarcinoma patients, help clinicians to assess the prognosis of patients and provide more basis for clinical treatment options.

## Conclusions

IVIM-DWI parameters of rectal adenoma reflect the degree of water molecule diffusion and microcirculation perfusion, providing quantitative information for clinical diagnosis. TA parameters can extract the multi-dimensional imaging features of selected tumors, better reflect the biological behavior and heterogeneity of tumors, and provide more information for differential diagnosis. The diagnostic efficacy of the combined prediction model of the two methods is superior to that of the single method, providing a more objective and accurate method for the prediction of EMVI,which can effectively make up for the deficiency of high-resolution MRI. Because it is important to accurately detect EMVI before treatment, it may affect the best treatment decisions, such as neoadjuvant therapy before operation. Our research may provide a better tool for clinical decision making.

## Data Availability Statement

The original contributions presented in the study are included in the article/**Supplementary Material**. Further inquiries can be directed to the corresponding authors.

## Author Contributions

Guarantor of integrity of the entire study: JD and TL. Study concepts and design: FG. Literature research: BS. Clinical studies: PW and XF. Experimental studies/data analysis: CW. Statistical analysis: BS. Manuscript preparation: FG. Manuscript editing: FG. All authors contributed to the article and approved the submitted version.

## Conflict of Interest

The authors declare that the research was conducted in the absence of any commercial or financial relationships that could be construed as a potential conflict of interest.

## Publisher’s Note

All claims expressed in this article are solely those of the authors and do not necessarily represent those of their affiliated organizations, or those of the publisher, the editors and the reviewers. Any product that may be evaluated in this article, or claim that may be made by its manufacturer, is not guaranteed or endorsed by the publisher.
