# Supplementary figures and images for: The Value of Intravoxel Incoherent Motion Diffusion-Weighted Magnetic Resonance Imaging Combined With Texture Analysis of Evaluating the Extramural Vascular Invasion in Rectal Adenocarcinoma
Source: Front Oncol. 2022 Mar 3;12:813138. doi: 10.3389/fonc.2022.813138 (PMC8927647; doi:10.3389/fonc.2022.813138)

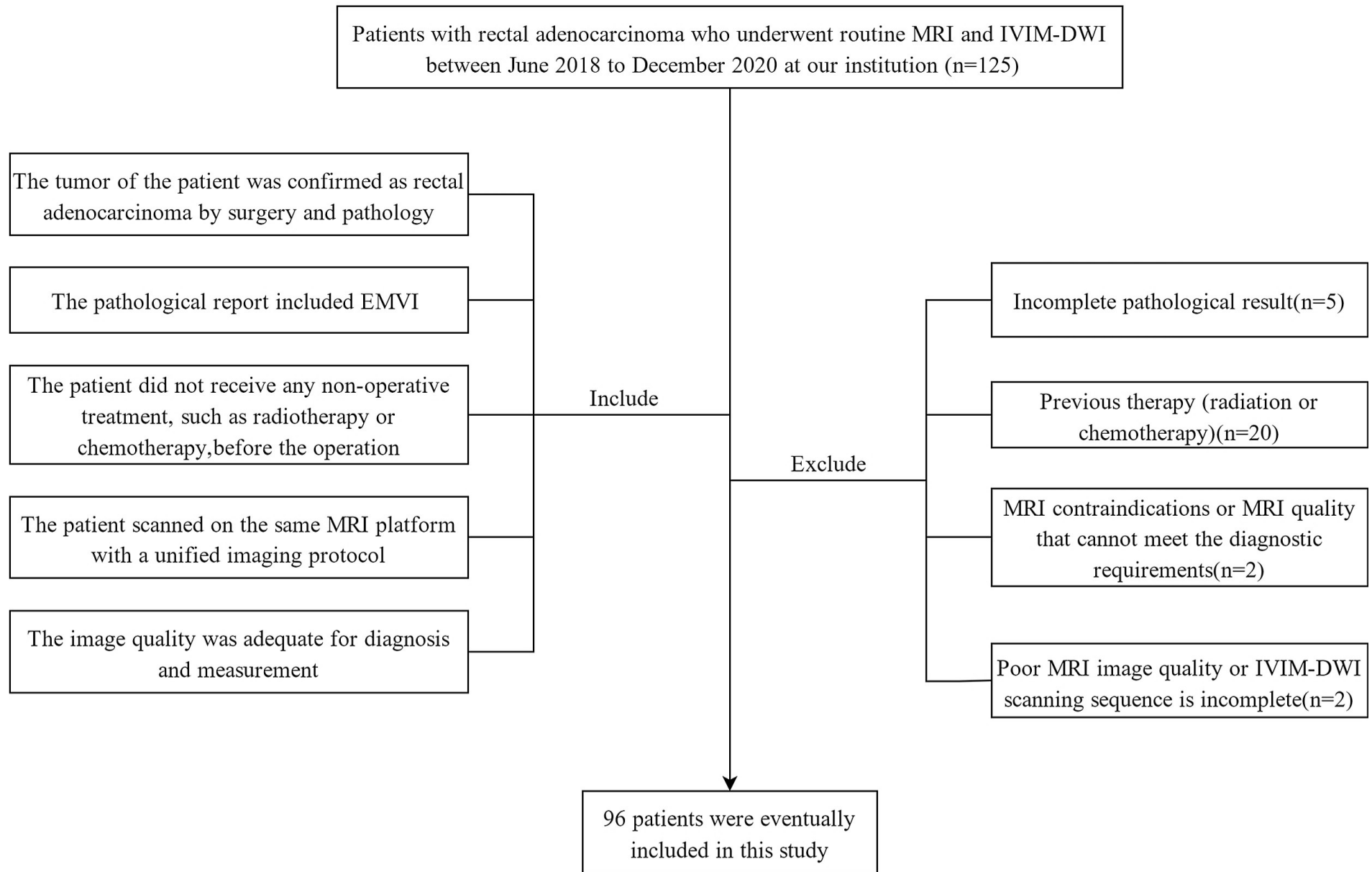

Supplement: Supplementary file 2 [file DataSheet_2.pdf]
